# The relationship between stromal cell derived SPARC in human gastric cancer tissue and its clinicopathologic significance

**DOI:** 10.18632/oncotarget.21133

**Published:** 2017-09-21

**Authors:** Yi Gao, Shui-Ping Yin, Xu-Shi Xie, Dan-Dan Xu, Wei-Dong Du

**Affiliations:** ^1^ Department of Pathology, Anhui Medical University, Hefei 230032, People's Republic of China; ^2^ Department of Urology, The First Affiliated Hospital of Anhui Medical University, Hefei 230022, People's Republic of China; ^3^ Department of Biology, School of Life Sciences, Anhui Medical University, Hefei 230032, People's Republic of China; ^4^ Department of Infectious Diseases, The Fourth Affiliated Hospital of Anhui Medical University, Hefei 230000, People's Republic of China

**Keywords:** SPARC, cancer-associated fibroblasts, tumor-associated macrophages, gastric cancer, tumor stroma

## Abstract

**Background:**

We aimed to investigate the cellular source of secreted protein acidic and rich in cysteine (SPARC) in gastric cancer tissues and the relationship between SPARC expression and its prognostic significance.

**Methods:**

The expression of SPARC in 365 primary advanced gastric adenocarcinomas and 39 non-cancerous tissues was evaluated by immunohistochemical staining. Double-immunofluorescence staining was used to reveal the cellular source of SPARC in tumor tissues. Western blotting and immunofluorescence staining were applied for verifying the endogenous expression of SPARC in human cell lines of gastric cancer and fibroblast.

**Results:**

Higher positivity of SPARC was observed in gastric cancer tissues than non-cancerous gastric tissues (P=0.000). The positivity of SPARC was related to age (P=0.032), tumor location (P=0.018), depth of tumor invasion (P=0.011), nodal metastasis (P=0.023), TNM stage (P=0.034), the differentiation degree (P=0.006) and pathological type (P=0.002) of gastric cancer. SPARC in gastric cancer tissues was mainly expressed by cancer-associated fibroblasts. SPARC also appeared in neovascular endothelial cells and a few tumor-associated macrophages. The endogenous expression of SPARC in fibroblasts was suppressed by mucus-producing gastric adenocarcinoma cells(MKN-45). Increased SPARC expression in gastric cancer tissue was suggestive of a shorter cumulative survival in the patients with gastric adenocarcinoma, though this difference was not statistically significant(P>0.05).

**Conclusion:**

SPARC in human gastric cancer tissue was derived from the stromal cells and was mainly produced by cancer-associated fibroblasts. Production of SPARC in fibroblasts was reduced by the mucus-producing gastric adenocarcinoma cells.

## INTRODUCTION

Secreted protein acidic and rich in cysteine (SPARC), known as basement-membrane protein 40 (BM-40) or Osteonectin, is an acidic extracellular matrix glycoprotein secreted by a variety of cells [1] and is associated with various physiological activities, including tissue repair and remodelling, extracellular matrix (ECM) composition, cell migration and apoptosis [2]. SPARC was found in various tumors and was expressed by either tumor cells or tumor stromal cells. However, its roles in tumor progression are quite controversial [3, 4]. SPARC acts as a tumor inhibitor in breast cancer, colorectal cancer, ovarian cancer, prostatic cancer and pancreatic cancer [5–11], but in glioma SPARC serves as a tumor promoter via enhancing tumor invasion and metastasis [12]. Gastric cancer is one of the leading causes of cancer-related mortality worldwide [13]. Significant up-regulation of the SPARC gene in gastric cancer tissue was first revealed by oligonucleotide microarray technology in 2002 [14]. The up-regulation of SPARC in gastric cancer tissue is not restricted to the gene level. Increased expression of SPARC protein in gastric cancer tissues has been proven by several independent groups [15–17]. By using a tissue microarray (TMA) of 436 gastric cancer samples and 92 non-cancerous tissue samples, Zhao et al. found that elevated SPARC expression was positively associated with age, tumor size, invasion depth, TNM stages and remote metastasis [16]. However, another study drew a different conclusion, which used conventional paraffin-embedded tissue sections of gastric cancer instead of a TMA from 65 gastric cancer patients [18]. It showed that elevated expression of SPARC was found in the early stage (I/II) and early tumor infiltration (T1/T2) as well as in patients without lymph node metastasis. These conclusions are controversial. Therefore, it is advisable to concurrently enlarge the observed size of tumor tissue sections and to increase the number of cases to evaluate the true role of SPARC in the progression of gastric cancer.

SPARC participates in tumor angiogenesis, migration, proliferation and survival through affecting growth factor signaling and cell-ECM interactions [4, 19]. However, the biological function of SPARC is versatile. SPARC derived from either tumor cells or tumor stromal cells plays various roles in tumor progression [4]. Therefore, determining the cellular sources of SPARC protein *in vivo* and *in vitro* is critical to understand the diverse role of SPARC in tumor progression of patients with gastric cancer.

## RESULTS

### Relationship between SPARC expression and the clinicopathologic characteristics of patients with gastric cancer

As summarized in Table 1, positive expression of SPARC protein was observed in 28.2% of the non-cancerous tissue sample (11 out of 39 individuals) and 63.3% of the gastric cancer tissue (231 out of 365 patients), respectively. The positivity of SPARC staining in gastric cancer tissues was significantly higher than that in non-cancerous tissues (Pearson’s Chi-square test, P=0.000, Table 1).

**Table 1:** Expression of SPARC in gastric cancer and non-cancerous tissues

	SPARC			*P*
	Negative (%)	Positive (%)	total	
Non-cancerous tissue	28(71.8%)	11(28.2%)	39	**0.000**
Cancer tissue	134(36.7%)	231(63.3%)	365	

SPARC positive staining was localized in the cytoplasm of tumor stromal cells with either moderate or strong staining. Correlation between SPARC expression and the clinicopathologic characteristics of the patients with gastric cancer were summarized in Table 2. Patients over 60 years old revealed a higher positivity of SPARC staining than patients below 60 years old (P=0.032). However, there was no statistical difference of the positivity of SPARC staining between male and female patients (P=0.454). Compared with other locations, gastric cancer tissue in the cardia and the fundus exhibited higher positivity of SPARC staining (P=0.018). In addition, the positivity of SPARC staining was related to nodal metastasis (P=0.023), TNM stage (P=0.034), pathohistological type (P=0.002) and differentiation degree (P=0.006). The size of primary tumor did not affect the positivity of SPARC staining in tumor samples (P=0.763), but the depth of tumor invasion exhibited a significant effect on the positivity of SPARC staining (P=0.011). As the invasion of the tumor from the mucosa to the serosa, higher positivity of SPARC was observed in the tumor tissue. However, once primary tumor invaded into the adjacent organs through the serosa, the positivity of SPARC was significantly decreased, instead. Lower positivity of SPARC was found in the gastric cancer patients with stage I than those with middle and late stages (II+III+IV) according to the TNM classification (P=0.034). Poorly differentiated gastric cancer tissues showed lower positivity of SPARC staining than well and moderately differentiated gastric cancer tissues(P=0.006). In addition, only 42.9% (24 out of 56 cases) of mucus-producing adenocarcinomas (e.g., signet ring cell and mucinous adenocarcinomas) were positive for SPARC staining, which was lower than any other pathohistological types of gastric cancer(P=0.002). The status of distant metastasis and cancer embolus in the stromal vessels did not affect the positivity of SPARC in gastric cancer tissue.

**Table 2:** Relationship between SPARC staining and clinicopathologic characteristics of gastric cancer

Clinical parameters	SPARC		
	Negative	Positive	P value
Age (y)			**0.032**
<60	61(43.6%)	79(56.4%)	
≥60	73(32.4%)	152(67.6%)	
Gender			0.454
Male	97(35.5%)	176(64.5%)	
Female	37(40.2%)	55(59.8%)	
Size(cm)			0.763
<5	66(37.5%)	110(62.5%)	
≥5	68(36.0%)	121(64.0%)	
Depth of invasion			**0.011**
T1	16(53.3%)	14(46.7%)	
T2	16(38.1%)	26(61.9%)	
T3	49(28.5%)	123(71.5%)	
T4	53(43.8%)	68(56.2%)	
Nodal metastasis			**0.023**
N0	49(35.8%)	88(64.2%)	
N1	26(31.7%)	56(68.3%)	
N2	21(29.2%)	51(70.8%)	
N3	38(51.4%)	36(48.6%)	
Distant metastasis			0.394
M0	118(37.6%)	196(62.4%)	
M1	16(31.4%)	35(68.6%)	
TNM stages			**0.034**
I	28(49.1%)	29(50.9%)	
II+III+IV	106(34.4%)	202(65.6%)	
Cancer embolus			0.217
Negative	105(35.2%)	193(64.8%)	
Positive	29(43.3%)	38(56.7%)	
Location			**0.018**
Cardia and fundus	60(31.1%)	133(68.9%)	
Other places	74(43.0%)	98(57.0%)	
Differentiation degree			**0.006**
Poor	75(42.9%)	100(57.1%)	
Moderate & well	59(31.1%)	131(68.9%)	
Pathohistologic type			**0.002**
Signet ring cell and mucinous adenocarcinoma	32(57.1%)	24(42.9%)	
Poorly differentiated adenocarcinoma	43(36.1%)	76(63.9%)	
Tubular and papillary adenocarcinoma	59(31.1%)	131(68.9%)	

### Staining characteristics of SPARC protein in gastric cancer

**Figure 1:**
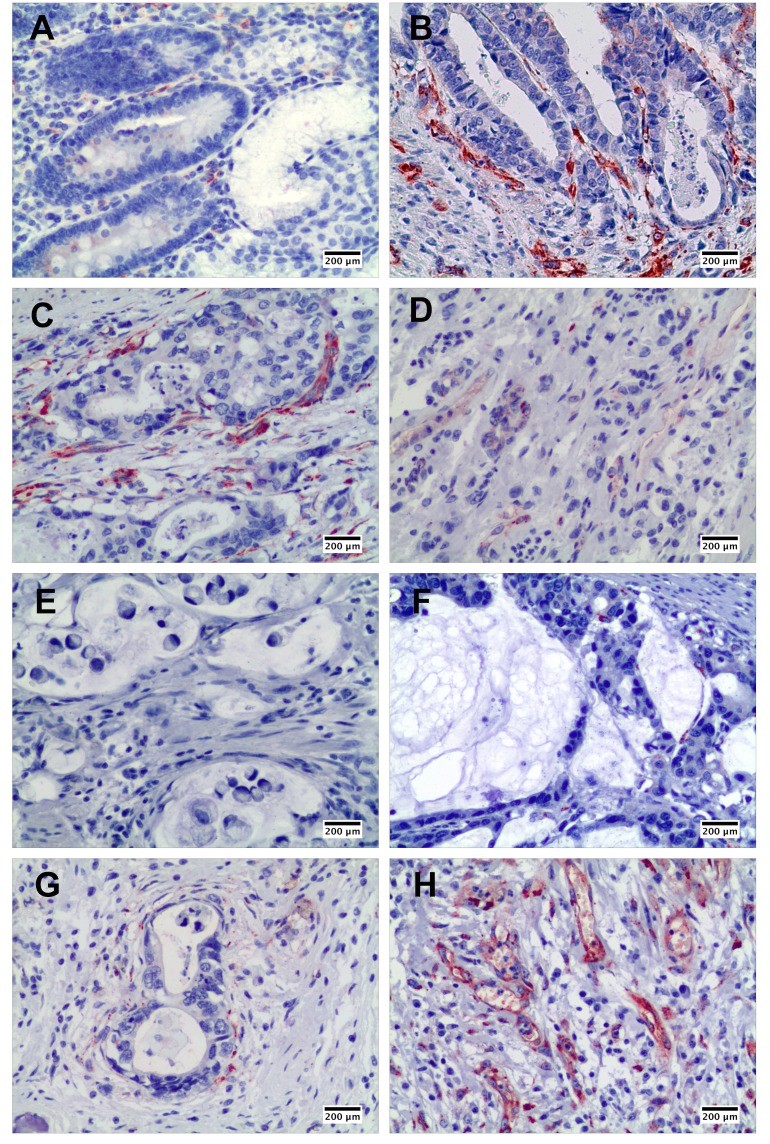
The expression of SPARC protein in human non-cancerous and gastric cancer tissues demonstrated by immunohistochemical staining SPARC expression in human non-cancerous tissues, scale bar: 200 μm **(A)**; in well differentiated papillary adenocarcinoma, scale bar: 200 μm **(B)**; in moderately differentiated tubular adenocarcinoma, scale bar: 200 μm **(C)**; in poorly differentiated adenocarcinoma, scale bar: 200 μm **(D)**; in signet ring cell carcinoma, scale bar: 200 μm **(E)**; in mucinous adenocarcinoma, scale bar: 200 μm **(F)**; around cancer nest, scale bar: 200 μm **(G)**; and in granulation tissue in gastric cancer tissue, scale bar: 200 μm **(H)**.

As shown in Figure 1, SPARC in the gastric cancer tissues was mainly expressed by stromal cells instead of cancer cells. Although a few SPARC positive cells were observed in the normal gastric mucosa (Figure 1A), much more SPARC positive cells were found in the interstitium of papillary adenocarcinoma (Figure 1B) and tubular adenocarcinoma (Figure 1C). Poorly differentiated gastric cancer revealed lower positivity of SPARC staining than well and moderately differentiated gastric cancers (Figure 1D). Furthermore, mucus-producing adenocarcinomas, including signet ring cell carcinoma (Figure 1E) and mucinous adenocarcinoma (Figure 1F), expressed much less SPARC protein compared with other pathohistological types of gastric cancer. Also, we observed that the distribution of SPARC positive cells in gastric cancer tissue was not equal. The stromal cells close to the cancer nests expressed more SPARC than those far away from the cancer nests (Figure 1G). The granulation tissue in gastric cancer samples showed dense and diffuse staining of SPARC protein. Abundant SPARC positive cells were located in the vascular structure of granulation tissue (Figure 1H).

**Figure 2:**
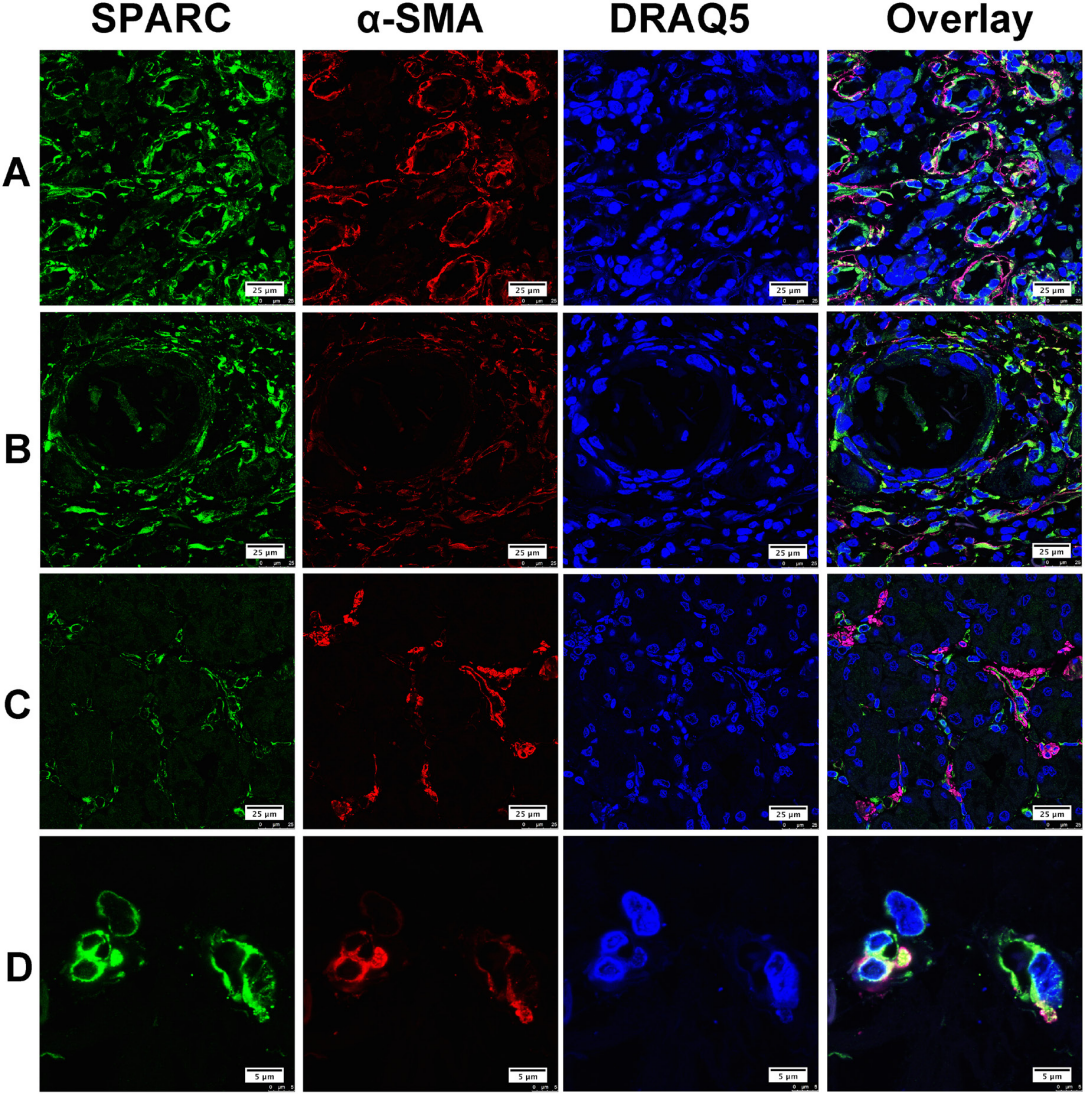
The expression of SPARC protein by cancer-associated fibroblasts in human gastric cancer tissues SPARC was detected by goat anti-human SPARC and Alexa Fluor 488-conjugated donkey anti-goat IgG secondary antibody (indicated in green). Cancer-associated fibroblasts were identified by mouse anti-alpha smooth muscle actin (α-SMA) antibody and Cy3-conjugated donkey anti-mouse IgG secondary antibody (indicated in red). Nuclei were stained with DRAQ5 (indicated in blue), and yellow staining indicates the co-localization of SPARC protein (green) and α-SMA (red). All pictures were taken at 63x magnification under an oil objective. The distribution of SPARC+/α-SMA+ cells in moderately differentiated tubular adenocarcinoma, scale bar: 25μm **(A)**; in well differentiated papillary adenocarcinoma, scale bar: 25μm **(B)**; in poorly differentiated adenocarcinoma, scale bar: 25μm **(C)** and in well differentiated adenocarcinoma, scale bar: 5 μm **(D)**.

Alpha smooth muscle actin (α-SMA), CD68 and CD31 were considered as the specific biomarkers of cancer-associated fibroblasts (CAF), tumor associated macrophages (TAM) and neovascular endothelial cells, respectively. In this study, double immunofluorescent staining was performed on five individual gastric cancer samples with representative pathohistological types, different lymph node metastatic stages (from N0-N3) and differentiation degrees. Confocal laser fluorescent microscopy was used to reveal the cellular source of SPARC protein in the gastric cancer tissues. SPARC was abundantly expressed by CAF in the tumor tissues as shown in Figure 2. The co-localization of SPARC and α-SMA varied greatly among the tumor tissue sections. In the Figure 2A, SPARC+/α-SMA+ cells were broadly observed. Figure 2B showed that both SPARC+/α-SMA+ and SPARC+/α-SMA- cells concurrently occurred. In the Figure 2C, SPARC and α-SMA were expressed by different cells in the tumor stroma. SPARC+/α-SMA+ cells were the most common phenotype in gastric cancer tissues compared with other phenotypes. Typical SPARC+/α-SMA+ cells were highlighted in the Figure 2D.

**Figure 3:**
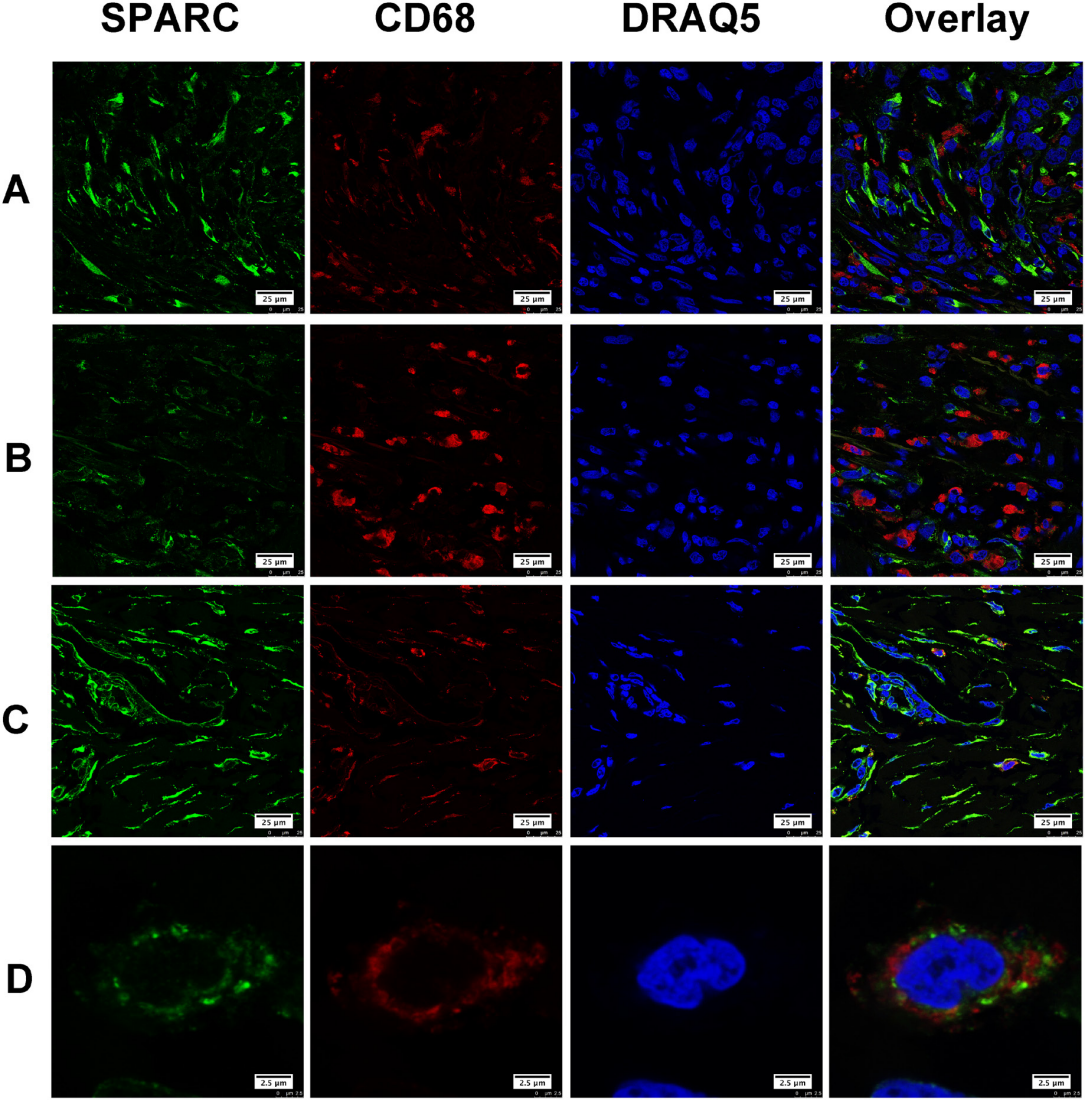
The expression of SPARC protein by tumor-associated macrophages in human gastric cancer tissues SPARC was detected by goat anti-human SPARC and Alexa Fluor 488-conjugated donkey anti-goat IgG secondary antibody (indicated in green). Tumor-associated macrophages were identified by mouse anti-human CD68 antibody and Cy3-conjugated donkey anti-mouse IgG secondary antibody (indicated in red). Nuclei were stained with DRAQ5 (indicated in blue), and yellow staining indicates the co-localization of SPARC protein (green) and CD68 (red). All pictures were taken at 63x magnification under an oil objective. The distribution of SPARC+/ CD68+ cells in moderately differentiated tubular adenocarcinoma, scale bar: 25 μm **(A)**; in poorly differentiated adenocarcinoma, scale bar: 25 μm **(B)**; in well differentiated papillary adenocarcinoma, scale bar: 25 μm **(C)**; typical SPARC+/CD68+ cells in well differentiated papillary adenocarcinoma, scale bar: 2.5 μm **(D)**.

**Figure 4:**
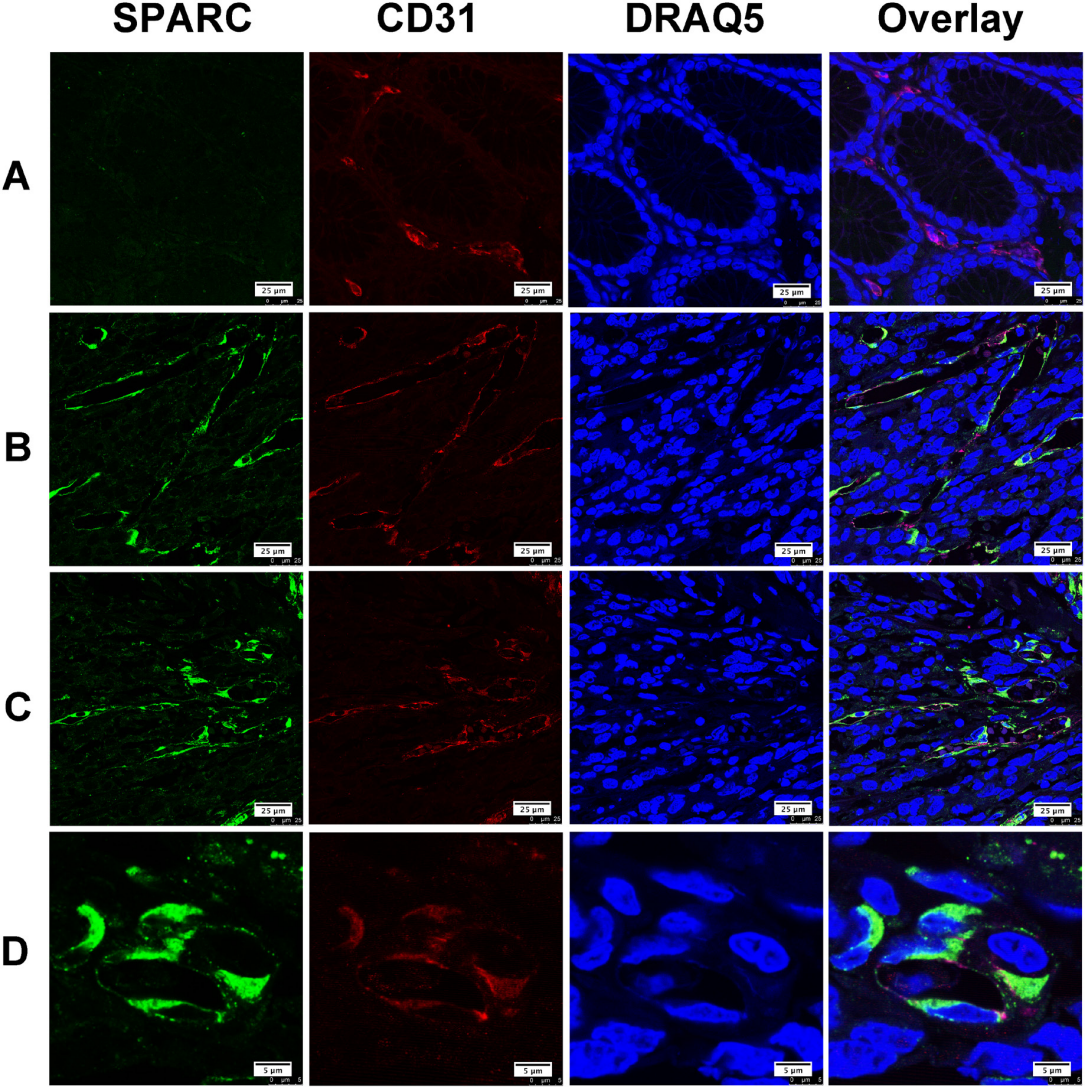
The expression of SPARC protein by vascular endothelial cells in human gastric cancer tissues SPARC was detected by goat anti-human SPARC and Alexa Fluor 488-conjugated donkey anti-goat IgG secondary antibody (indicated in green). Vascular endothelial cells were identified by mouse anti-human CD31 antibody and Cy3-conjugated donkey anti-mouse IgG secondary antibody (indicated in red). Nuclei were stained with DRAQ5 (indicated in blue), and yellow staining indicates the co-localization of SPARC protein(green) and CD31 (red). All pictures were taken at 63x magnification under an oil objective. The distribution of SPARC+/CD31+ cells in noncancerous tissue, scale bar: 25μm **(A)**; in well differentiated papillary adenocarcinoma, scale bar: 25μm **(B)**; in poorly differentiated adenocarcinoma, scale bar: 25 μm **(C)**; in moderately differentiated tubular adenocarcinoma, scale bar: 5μm **(D)**.

Gastric cancer tissues selected for double immunofluorescent staining showed wide infiltration of tumor-associated macrophages (TAM), which were characterized by CD68. We found that only a few CD68 positive TAM appeared in the area with abundant SPARC positive cells. Most of TAM did not express SPARC protein (Figure 3A). Similarly, SPARC positive cells were seldom observed in the area abundantly infiltrated by TAM (Figure 3B). We did observe a few CD68 positive cells expressing SPARC (Figure 3C). Unlike the cellular distribution of SPARC in CAF, SPARC appeared in the cytoplasm of a few TAM in the form of scattered particles (Figure 3D). In non-cancerous gastric tissues, SPARC did not appear in CD31-positive endothelial cells of the capillaries (Figure 4A). However, in gastric cancer tissues, SPARC protein was highly co-localized with CD31 in the neo-vessels (Figure 4B-4D).

### Endogenous SPARC expression in fibroblasts and gastric cancer cell lines

**Figure 5:**
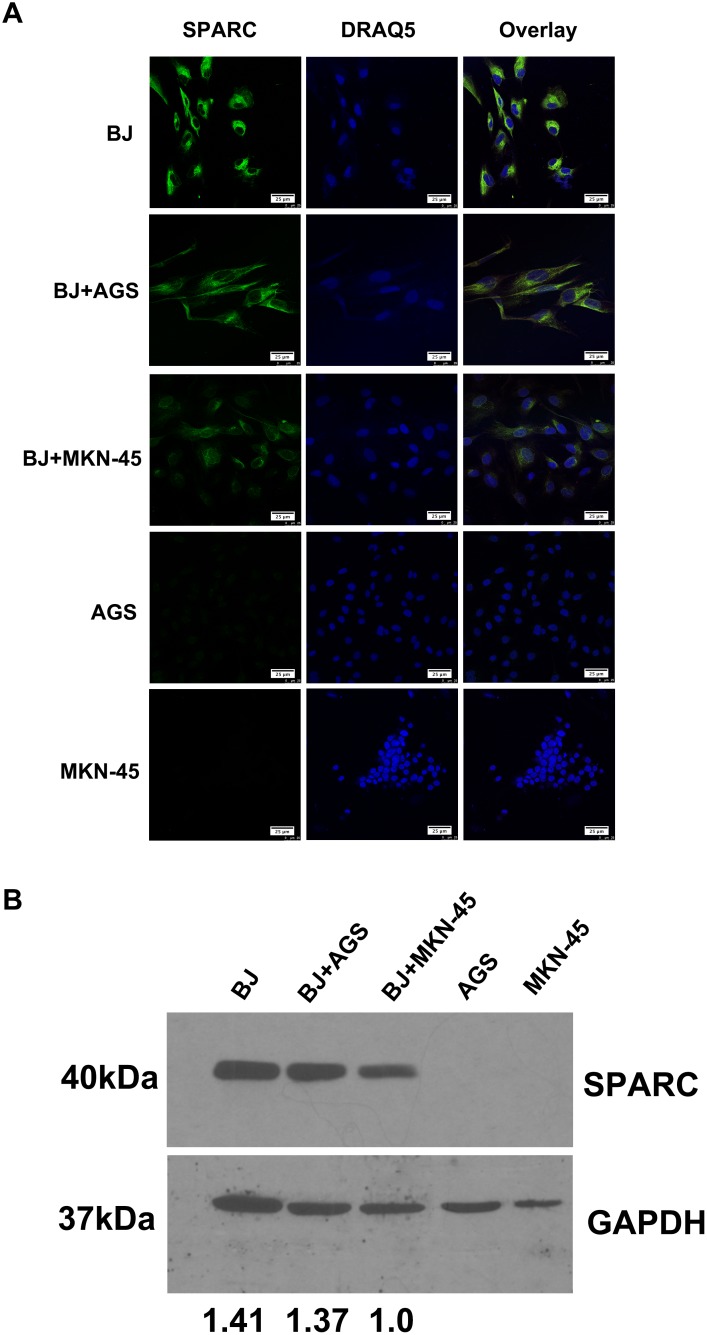
The expression of SPARC protein in a co-culture model of human fibroblasts and gastric cancer cells **(A)** The expression of SPARC protein in human fibroblast cells (BJ) and gastric cancer cells (AGS, MKN-45) was visualized by immunofluorescent staining. SPARC was detected by goat anti-human SPARC and Alexa Fluor 488-conjugated donkey anti-goat IgG secondary antibody (indicated in green). Nuclei were stained with DRAQ5 (indicated in blue). All pictures of immunofluorescence staining were taken at 63x magnification under an oil objective, scale bar: 25 μm. **(B)** The expression of SPARC protein in human fibroblast cells (BJ) and gastric cancer cells (AGS, MKN-45) was visualized by western blotting. SPARC protein in human fibroblast cells (BJ) and gastric cancer cells (AGS, MKN-45) were demonstrated by probing the cell lysates with goat anti-human SPARC antibody (AF941, R&D system), molecular weight: 40kDa. GAPDH was tested in the same gel as internal control for the loading sample, molecular weight: 37kDa. The relative abundance of SPARC expression in different lanes was demonstrated via normalization with the expression of GAPDH. BJ, AGS and MKN-45 meant that the cells were cultured alone for 48h; BJ+AGS and BJ+MKN-45 meant that BJ cells were co-cultured with AGS cells or BJ+MKN-45 cells for 48h, respectively.

Similar to the previous investigation [20], in this study abundant SPARC was observed in human fibroblast cell line instead of gastric cancer cells (Figure 5A). No endogenous SPARC protein was found in gastric cancer cell lines (AGS and MKN-45). A co-culture system was used to test whether gastric cancer cells affected the SPARC expression in fibroblasts. As demonstrated in the Figure 5B, the endogenous expression of SPARC in fibroblasts was suppressed while co-cultured with MKN-45 cells, which were derived from human mucus-producing gastric adenocarcinoma.

### Relationship between SPARC expression and cumulative survival

**Figure 6:**
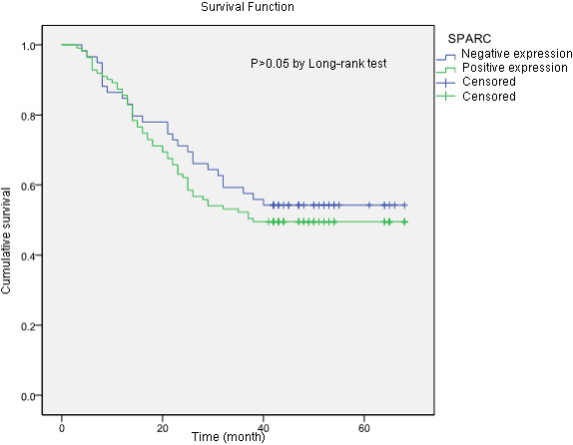
The Kaplan-Meier survival curves of two groups of gastric cancer patients according to the tissue expression of SPARC protein The samples with more than 10% of the stromal cells stained either moderately or strongly for SPARC was included in the SPARC positive group. The estimated survival time of patients in SPARC positive group was shorter than that in the SPARC negative group (42.7 months vs 45.4 months), but the difference was not statistically significant (P>0.05).

Eighty-three patients died of tumor progression during the follow-up period. The mean survival time was 43.6 months (95% confidence interval ranged from 39.8 to 47.5 months). The estimated survival time of gastric cancer patients without SPARC expression was 45.4 months, which was longer than patients in the SPARC-positive group (42.7 months). However, the difference was not statistically significant (P>0.05). The cumulative survival results suggested that the SPARC expression in tumor microenvironment would not significantly affect the prognosis of the patients with gastric cancer (Figure 6).

## DISCUSSION

Cell sources of SPARC differed in various tumor tissues. In malignant pleural mesothelioma, both tumor cells and tumor stromal cells expressed SPARC. Increased serum level of SPARC predicted poor prognosis of the patients [21]. In malignant ovarian, colorectal and gastric cancers, SPARC was mainly derived from tumor stromal cells [4, 16, 22]. In melanoma and breast cancer tissues, tumor cells were the main source of SPARC [22, 23]. In this study, we observed that SPARC protein was solely expressed by the stromal cells of gastric cancer tissue instead of cancer cells. *In vitro* western blotting and immunofluorescence staining further demonstrated that human gastric cancer cells (MKN-45 and AGS) did not produce SPARC. Previous research based on immunohistochemical staining and morphological evaluation assumed that tumor stromal fibroblasts were important source of stroma-based SPARC [21]. By using confocal laser scanning microscopy and specific biomarkers for CAF, TAM and vascular endothelial cells, in the study we proved that SPARC in gastric cancer tissues was mainly expressed by cancer-associated fibroblasts. SPARC also appeared neovascular endothelial cells and a few tumor-associated macrophages.

CAF are characterized by the abundant expression of α-SMA [24]. Alpha-SMA positive CAF predicted poor prognosis in patients with colorectal cancers [25]. CAF may influence the cellular microenvironment by releasing ECM proteins, growth factors, proteases, cytokines, and chemokines [24]. SPARC expressed by CAF might act as a tumor inhibitor during the early stage since SPARC protein can induce apoptosis in tumor cells [26]. It was supposed that gastric cancer cells could escape the nonspecific apoptosis induced by CAF through suppressing the endogenous expression of SPARC in fibroblasts. In gastric cancer tissue, most of TAM did not express SPARC. A few TAM showed positive SPARC in the form of scattered particles, which indicated that these SPARC particles might enter cells by the process of phagocytosis of TAM. The role of TAM in scavenging SPARC has been investigated *in vivo* and *in vitro*. Reduced exposure of tumor cells to SPARC facilitates tumor growth process [26]. As shown in Figure 4, SPARC was not expressed by CD31-positive cells in normal gastric tissue but was detected in the neovascular endothelial cells of gastric cancer tissue, suggesting that neovascular endothelial cells would be an additional source of SPARC in gastric cancer tissue.

It was reported that gene level of SPARC in gastric cancer tissues was increased, which was associated with tumor TNM stage, lymph node metastasis and long-term survival [14, 15, 20, 27]. In this study, 365 gastric cancer patients and 39 non-cancerous samples were enrolled to investigate the positivity of SPARC protein on the large tissue sections instead of using tissue microarray. We found that the positivity of SPARC protein in gastric cancer tissues was much higher than that in non-cancerous tissues, which is also reported by other research [16, 17]. Positive SPARC expression was identified in 63.3% of gastric cancer tissues and 28.2% of non-cancerous tissues. As the depth of tumor invasion and the number of metastatic lymph nodes increased, more SPARC positive cells in the gastric cancer tissue were found. However, this trend changed when the tumor progressed to later stages. Increased infiltration of fibroblasts during the early stage of gastric cancer was thought as a host defence mechanism to restrict tumor progression. The dynamic change of SPARC protein expression in gastric cancer tissue might reflect the real activity of fibrosis in anti-tumor process. Signet ring cell carcinoma and mucinous adenocarcinoma are characterized by the loss of cell-cell adhesion molecules and the accumulation of mucin in the cytoplasm, respectively [28]. In this study, fewer SPARC positive cells were observed in the signet ring cell carcinoma and mucinous adenocarcinoma tissues compared with other pathohistological types of gastric cancer. *In vitro* experiments indicated that human gastric cancer cells, especially human mucus-producing gastric adenocarcinoma derived cells (MKN-45), could potentially inhibit the expression of SPARC in fibroblasts (Figure 5B). Normal production of mucin by the gastric mucosa was effective in preventing *helicobacter pylori* infection and suppressing tumor-promoting inflammation. However, overproduction and accumulation of mucin in the extracellular matrix of tumor tissue would affect the infiltration of immune cells and the early wound repair reaction upon initiation of local carcinogenesis [28]. In this study, we observed that there was higher positivity of SPARC staining in the proximal stomach (cardia and fundus) than in the middle and the distal regions of the stomach. We supposed that it might result from the increased gastroesophageal reflux in the process of gastric cancer. The long-term exposure of gastroesophageal reflux led to chronic damage of mucosa and wound repair by fibroblasts in tumor microenvironment.

Even though previous researches proved that high expression of SPARC gene in gastric cancer samples was correlated with poor patient survival [15, 29], our study did not reveal any effect of SPARC protein on the overall survival of the patients during our follow-up. The deviation between the researches might be attributed to the differences in tumor tissue structures or tissue section sizes and quantitative methods for immunohistochemical staining. In this study, we used conventional large tissue sections of gastric cancer instead of TMA for staining of SPARC and counting of positive cells since we found that the distribution of SPARC positive cells in the tumor tissues was unequal. More SPARC positive cells were located in the stroma adjacent to the cancer nest (Figure 1). Increased tissue sizes of gastric cancer would be able to offer a larger spectrum to reveal how the relationship of the parenchyma and the stroma of the tumor was.

In conclusion, our data demonstrated that gastric cancer stromal tissue expressed higher positivity of SPARC protein. CAF were the main source of SPARC in gastric cancer tissue. The expression of SPARC protein in CAF was suppressed by mucus-producing gastric cancer cells. This research on SPARC expression in cancer-associated fibroblasts provided new insight into biological and functional studies of SPARC as well as potential clinical treatment of gastric cancer.

## MATERIALS AND METHODS

### Patients and tissue samples

Three hundred and sixty-five Chinese patients with primary advanced gastric adenocarcinoma and thirty-nine non-cancerous patients were enrolled in this study. The patients underwent a gastrectomy at the Department of Gastrointestinal Surgery, The First Affiliated Hospital of Anhui Medical University of China between September 2009 and September 2012 and had not received any preoperative chemotherapy or radiotherapy. Tumor tissue samples were obtained from the patients. Basic clinical and pathological features of the patients, such as age, gender, tumor location, tumor size, and distant metastasis were retrospectively collected. The depth of invasion, nodal metastasis, cancer embolus, differentiation degree, pathohistological type and TNM stage of the tumor were evaluated by two independent pathologists according to the seventh American Joint Committee. Survival follow-up was successfully obtained in 170 of the 365 patients until November 2015. This study was approved by the Ethics Committee of the Anhui Medical University (Nr. 20080253).

### Immunohistochemistry and evaluation

Immunohistochemical staining for SPARC was performed on formalin-fixed, paraffin-embedded tissue sections (4μm thick) of the primary gastric cancers. The sections were incubated with goat anti-human SPARC/osteonectin antigen affinity-purified polyclonal antibody (Catalogue # AF941, dilution 1:60, R&D systems, Minneapolis, MN, USA) at 3μg/ml. Approximately 100μl of the diluted primary antibody was added to each section. HRP-conjugated donkey anti-goat IgG antibody (Catalogue #150430, dilution 1:400, Dianova, Hamburg, Germany) was diluted in 1% BSA dissolved in PBS buffer (1x PBS buffer, pH 7.4, Gibco), and 100μl of the diluted secondary antibody was added to each section. SPARC staining was evaluated on an Olympus CX31 microscope (Olympus, Center Valley, PA) by two independent pathologists who were blinded to each other’s findings and five random views in the area of tumor stroma were taken for counting. Positive SPARC staining was localized in the cytoplasm of stromal cells. The section was considered to be positive if more than 10% of tumor stromal cells in the whole section showed either moderate or strong SPARC staining [30].

### Immunofluorescence staining

Immunofluorescence staining for SPARC was performed on formalin-fixed, paraffin-embedded tissue sections (4μm thick) which were selected from five primary advanced gastric cancers with representative pathohistological types and various status of local nodal metastasis (from N0 to N3), including 1 case of well differentiated papillary adenocarcinoma, 3 cases of moderately differentiated tubular adenocarcinoma and 1 case of poorly differentiated adenocarcinoma. The primary antibodies used were listed as following: goat anti-human SPARC polyclonal antibody (Catalogue #AF941, dilution 1:60, R&D, Minneapolis, MN, USA), mouse anti-human CD31 monoclonal antibody (Catalogue # IR61061, dilution 1:15, Dako, Santa Clara, CA, USA,), mouse anti-human CD68 monoclonal antibody (Catalogue #MSK055 KP-1, dilution 1:50, Zytomed, Berlin, Germany) and mouse anti-smooth muscle actin monoclonal antibody (Catalogue #MSK030-05, dilution 1:100, Zytomed, Berlin, Germany). Primary antibodies were diluted in 1% BSA dissolved in PBS buffer, and 100 μl of the diluted primary antibody mixture was added to each section. The secondary antibodies used were listed as following: Alexa Fluor 488-conjugated donkey anti-goat IgG (Catalogue #705-546-147, dilution 1:400, Dianova, Hamburg, Germany), Cy3-conjugated donkey anti-mouse IgG (Catalogue #715-165-151, dilution 1:400, Dianova, Hamburg, Germany), Cy3-conjugated donkey anti-rabbit IgG (Catalogue #715-165-152, dilution 1:400, Dianova, Hamburg, Germany) and DRAQ5 (Catalogue #4084, dilution 1:1000, Cell Signaling Technology, Danvers, MA, USA). The secondary antibodies were prepared in 1% BSA dissolved in PBS buffer, and 100μl of the diluted secondary antibody mixture was added. Sections were kept in a dry and dark environment. The co-localization of SPARC with other biomarkers was demonstrated by Confocal laser scanning microscopy using a Leica SP8 Microscope.

### Cell line culture and immunofluorescence staining

Human foreskin-derived fibroblasts (BJ) and human gastric adenocarcinoma cell lines, AGS and MKN-45, were purchased from ATCC (USA). BJ and AGS cells were maintained in DMEM GlutaMAX (Invitrogen, ThermoFisher Scientific, Germany) supplemented with 10% fetal bovine serum and 1% penicillin/streptomycin. MKN-45 cells were maintained in RPMI 1640 medium (Invitrogen, ThermoFisher Scientific, Germany) supplemented with 10% fetal bovine serum and 1% penicillin/streptomycin.

BJ cells (1×10^4^) and human gastric adenocarcinoma cells (AGS and MKN-45) were separately seeded in 24-wells plates with one 22×22 mm coverslip per well. After cultured for 12 hours, one coverslip with BJ cells and another coverslip with either AGS or MKN-45 were transferred into a single well of a 6-wells plate and maintained in 2 ml of DMEM complete medium for 48 hours. Cells on each coverslip were fixed with 4% paraformaldehyde and prepared for immunofluorescence staining. All pictures of immunofluorescence staining were taken at 63x magnification under an oil objective.

### Western blotting

Cells on the top of the coverslip as described above were harvested by adding 500 μl of 0.05% trypsin/EDTA solution (Gibco). 1×10^6^ fibroblasts (BJ) and human gastric adenocarcinoma cells (AGS or MKN-45) were re-suspended in 200μl of 2x Laemmli sample buffer (Bio-Rad, 1610737) supplemented with 2-mercaptoethanol. From this, 50 μl of the cell lysates were loaded into an SDS-PAGE gel and transferred to nitrocellulose membranes (Protran). The following antibodies were used: goat anti-human SPARC (R&D systems, AF941), rabbit anti-GAPDH (ab9485, Abcam), HRP-labelled mouse anti-goat IgG (Dianova) and donkey anti-rabbit IgG (Amersham Biosciences). The membrane was developed using the X-ray film processor CAWOMEN 2000 IR after adding Luminata Forte Western HRP substrate (Merck, Millipore) to the membranes. GAPDH was tested in the same gel as internal control for the loading sample. The relative abundance of SPARC expression in different lanes was demonstrated after normalization with the expression of GAPDH.

### Statistical analysis

The analysis was performed with SPSS 19.0 (SPSS, Chicago, IL, USA). Pearson’s Chi-square test was used to compare the SPARC expression in gastric cancer and non-cancerous tissue. Pearson’s Chi-square test was also used to compare clinicopathologic variables and SPARC expression. A Kaplan–Meier analysis was used to evaluate the cumulative survival time. All statistical analyses were two-sided, and P<0.05 was considered statistically significant. The log-rank test was used to compare the prognostic significance of the individual variables on survival.

## Abbreviations

SPARC: secreted protein acidic and rich in cysteine
TAM: tumor-associated macrophages
CAF: cancer-associated fibroblasts
ECM: extracellular matrix
α-SMA: alpha-smooth muscle actin
TMA: tissue microarray
